# Evaluating Prostate Cancer Using Fractional Tissue Composition of Radical Prostatectomy Specimens and Pre-Operative Diffusional Kurtosis Magnetic Resonance Imaging

**DOI:** 10.1371/journal.pone.0159652

**Published:** 2016-07-28

**Authors:** Edward M. Lawrence, Anne Y. Warren, Andrew N. Priest, Tristan Barrett, Debra A. Goldman, Andrew B. Gill, Vincent J. Gnanapragasam, Evis Sala, Ferdia A. Gallagher

**Affiliations:** 1 Department of Radiology, University of Cambridge, Addenbrooke’s hospital, Cambridge, United Kingdom; 2 Department of Radiology, Memorial Sloan Kettering Cancer Center, 1275 York Ave, New York, NY, United States of America; 3 Department of Histopathology, University of Cambridge, Addenbrooke’s hospital, Cambridge, United Kingdom; 4 Department of Epidemiology & Biostatistics, Memorial Sloan Kettering Cancer Center, 1275 York Ave, New York, NY, United States of America; 5 Department of Urology, University of Cambridge, Addenbrooke’s hospital, Cambridge, United Kingdom; Rush University Medical Center, UNITED STATES

## Abstract

**Background:**

Evaluating tissue heterogeneity using non-invasive imaging could potentially improve prostate cancer assessment and treatment.

**Methods:**

20 patients with intermediate/high-risk prostate cancer underwent diffusion kurtosis imaging, including calculation of apparent diffusion (D_app_) and kurtosis (K_app_), prior to radical prostatectomy. Whole-mount tissue composition was quantified into: cellularity, luminal space, and fibromuscular stroma. Peripheral zone tumors were subdivided according to Gleason score.

**Results:**

Peripheral zone tumors had increased cellularity (p<0.0001), decreased fibromuscular stroma (p<0.05) and decreased luminal space (p<0.0001). Gleason score ≥4+3 tumors had significantly increased cellularity and decreased fibromuscular stroma compared to Gleason score ≤3+4 (p<0.05). In tumors, there was a significant positive correlation between median K_app_ and cellularity (ρ = 0.50; p<0.05), and a negative correlation with fibromuscular stroma (ρ = -0.45; p<0.05). In normal tissue, median D_app_ had a significant positive correlation with luminal space (ρ = 0.65; p<0.05) and a negative correlation with cellularity (ρ = -0.49; p<0.05). Median K_app_ and D_app_ varied significantly between tumor and normal tissue (p<0.0001), but only median K_app_ was significantly different between Gleason score ≥4+3 and ≤3+4 (p<0.05).

**Conclusions:**

Peripheral zone tumors have increased cellular heterogeneity which is reflected in mean K_app_, while normal prostate has a more homogeneous luminal space and cellularity better represented by D_app_.

## Introduction

Prostate cancer (PCa) is the most common male cancer in the US and Europe and the incidence is increasing [1]. The routine clinical assessment of PCa involves histopathological evaluation following biopsy to determine a Gleason score (GS), which is primarily based on tissue architecture rather than cytological features [2,3]. This score can predict disease aggressiveness and treatment failure [4,5]. Unfortunately, accurate pre-treatment assessment of PCa aggressiveness remains difficult due to the limitations of biopsy, including sampling error [6,7]. There has been recent interest in combining information from histopathology with imaging, as well as other tissue-based biomarkers, to allow for better disease assessment [8,9].

Magnetic resonance imaging (MRI) is the main imaging modality for the diagnosis, staging and determination of treatment response in prostate cancer. Clinical MRI routinely incorporates diffusion weighted imaging (DWI), which detects the molecular movement of water in tissue, and how this is altered in the highly cellular tumor environment [10–15]. Quantitative parameters can be obtained from a series of diffusion-weighted MRI acquisitions at different diffusion gradient strengths by fitting to a mathematical model used to describe the decay of measured MRI signal with increasing amounts of applied diffusion weighting or b-value. Diffusional kurtosis imaging (DKI) is an extension of DWI that attempts to further quantify water diffusion by evaluating non-Gaussian diffusion within each voxel [16]. When applied to DWI, kurtosis theoretically reflects how the presence of cells and the heterogeneous nature of tissue structure may distort the normal distribution of water diffusion. DKI derives two quantitative metrics: the “apparent diffusion coefficient” (D_app_) and a unitless “apparent kurtosis” parameter (K_app_). The role of DKI in PCa has been evaluated previously in retrospective studies, and the results suggest a possible correlation between K_app_ and disease aggressiveness [17–20]. However evaluation of the biophysical basis of DKI and its correlation to the changes in tissue composition that occur in prostate cancer has been limited. Therefore, the objective of this study was: to assess the histological tissue composition of normal tissue and tumors of different grade from the peripheral zone (PZ) of the prostate, and to correlate these variations in composition with non-invasive imaging of water diffusion pre-prostatectomy using the apparent diffusion (D_app_) and apparent kurtosis (K_app_) parameters derived from DKI.

## Materials and Methods

This study was a prospective single-institution trial, approved by the local institutional review board (IRB) and ethics committee (Cambridgeshire 10/H0304/54). Informed written patient consent was obtained on a paper consent form. This consent procedure was approved by the ethics committee.

Consecutive eligible patients were approached and twenty-six patients with PCa were enrolled between July 2011 and April 2012. Inclusion criteria were: (1) biopsy-proven prostate cancer considered intermediate/high risk using the D’Amico risk classification system [21]; (2) radical prostatectomy planned; (3) needle-biopsy performed at least 6 weeks before study MRI to reduce the effects of hemorrhage. Exclusion criteria were: (1) severe artifact on DWI; (2) significant tumor (defined as > 0.5 cm^3^) present only in the transition zone. 6 patients were excluded because of substantial susceptibility artifact on DWI (n = 1) or because significant cancer was not present in the PZ (n = 5). Therefore the final study population constituted 20 patients.

### MR imaging

Diffusion-weighted echo-planar imaging was acquired with a 3 Tesla Signa HDx scanner (GE Healthcare, Waukesha, WI) using an 8-channel phased array coil. Imaging parameters were as follows: echo/repetition time 89/5000 ms; 8 or more signal averages; field-of-view 32x32 cm^2^; matrix 256x256; slice thickness was 4 mm with a gap between slices of 1 mm; parallel imaging factor of 2; acquisition time of 6 minutes 45 seconds. The use of echo-planar imaging, in conjunction with parallel imaging, allows for rapid data acquisition significantly reducing artifact due to motion, blurring, and distortion [22, 23]. The b-value of a DWI or DKI acquisition measures the degree of diffusion weighting applied. For this study b-values of 150, 600, 1050 and 1500 s/mm^2^ were used. The b-values were chosen to include a non-zero low b-value (150 s/mm^2^), two additional b-values evenly spaced between 150 s/mm^2^ and 1500 s/mm^2^ (600, 1050 s/mm^2^), as well as a high b-values of 1500 s/mm^2^. A b-value of 0 s/mm^2^ was not included as studies have shown that low b-values can be susceptible to pseudoperfusion bias which can be avoided by using a low b-value >100 [24]. The upper range of the b-values was determined to allow for successful DKI parameter calculation, while also seeking to minimize the biasing/confounding effects of noise on high b-value images. At least 3 separate b-value acquisitions are required for DKI fitting. Axial T_1_-weighted (T_1_W) images and high-resolution T_2_-weighted (T_2_W) images of the pelvis were acquired in axial, sagittal and coronal planes. Noise compensation was performed through acquisition of a noise-only image by using the identical acquisition and reconstruction without using the usual radiofrequency excitation pulses. The DKI model used a second-order approximation to the exponential dependence of DWI signal *S* with b-value (*b*):

$${S=S}_{0}*\text{exp}\left({\text{-bD}}_{\text{app}}+1/6*{\text{b}}^{2}{\text{D}}_{\text{app}}{}^{2}{\text{K}}_{\text{app}}\right)$$

where *S*_*0*_ is the signal when *b* = 0 s/mm^2^, D_app_ is the apparent diffusion coefficient, and K_app_ is the apparent diffusional kurtosis. Quantitative maps of D_app_ and K_app_ were calculated using custom software written in Matlab (Mathworks, MA, USA), with the measured signals for each pixel against b-value fitted to the above equation by non-linear fitting using the trust-region reflective algorithm. For noise compensation, the noise parameter (n) was measured from the mean of the noise-only images (after smoothing with a 25×25 pixelwise adaptive Wiener filter) divided by $\sqrt{2/\pi }$ and the data was fitted to the expected signal biased by noise (S_n_) which is given by the root mean square of the modeled signal without noise (S) at the measured noise level, according to the following equation:

$${\text{S}}_{\text{n}}={\left({\text{S}}^{2}+{\text{n}}^{2}\right)}^{1/2}$$

### Histopathology assessment and comparison with imaging

Following surgery, each *ex vivo* prostate was fixed in formalin and processed according to international recommendations [25]. The specimen was measured in three dimensions and was oriented by the location of the seminal vesicles, posterior surface of the prostate, and by the position of the urethra. The surgical margins were inked. The apical end and basal cone were amputated, sliced from left to right into 4 mm thick pieces, and placed in small cassettes preserving their order. The remaining gland was cut transversely into 5 mm whole-mount parallel slices in the horizontal plane from inferior to superior. A cutting guide was used to hold the prostate so that there was neither tissue loss nor distortion when slicing into the transverse slices. Packing material was also added to the cassettes to avoid distortion of the large slices during standard tissue processing. Five-micron sections were taken from each of the 5 mm whole-mount parallel slices for histopathologic analysis. Tumor was outlined on hematoxylin and eosin (H&E) stained sections from each slice by an experienced uropathologist specializing in PCa (> 10 years of experience in uropathology). Each slice was manually co-registered to a corresponding MR image, allowing for the effects of tissue processing if necessary, based on the location of anatomical features (prostatic hyperplasia nodules, ejaculatory ducts, urethra), the relative diameter of the prostate, and the approximate distance from the base or apex. Two radiologists (2 and 7 years of experience with prostate MRI) in consensus determined tumor location on MRI by matching T_2_W MRI with the histopathological slides and transferring the outline of the largest PZ tumor on histology to the D_app_ maps. A second equivalent outline was drawn in the contralateral normal PZ.

### Image segmentation

**Fig 1:**
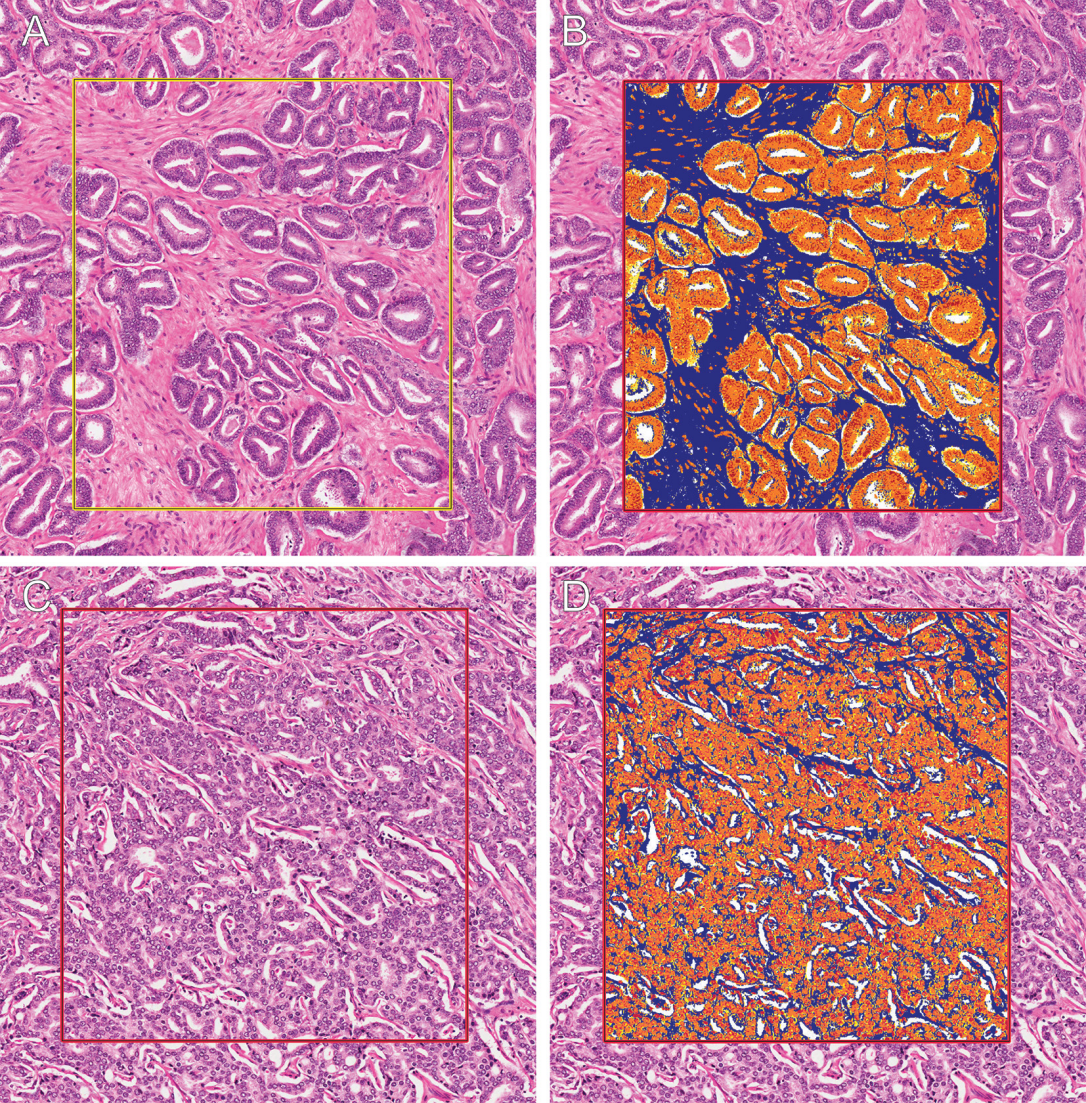
Digitalized prostate histopathology. **A, B**: Histologic prostate tissue sections (x10 objective) from regions of PZ tumor tissue with a Gleason grade of 3. Each H&E stained whole-mount section was digitalized at x20 resolution (A). The digitalized slide was then segmented, using a positive pixel counting algorithm, into three main tissue components (B): cellularity (orange and yellow), fibromuscular stromal matrix (FSM, blue), and luminal space (white). This Gleason grade 3 region has limited luminal space and is highly cellular; the malignant glandular architecture is infiltrating into the surrounding FSM. **C**, **D**: The H&E stained whole-mount section from a primarily Gleason grade 4 region (C) was also digitalized at x20 resolution and segmented (D). This Gleason grade 4 region shows complex architecture with limited luminal space and malignant cellularity, which has extensively infiltrated and almost completely replaced the FSM.

The histopathologist reviewed the digitalized whole-mount slides (20x magnification) and digitally outlined regions of prostate cancer and normal prostate tissue to match the previously established radiologic and pathologic regions of interest. Tumors were categorized into two categories of aggressiveness according to GS: GS ≤ 3+4 and GS ≥ 4+3. This distinction effectively subdivides GS 7 disease according to the primary Gleason grade and has been shown previously to be clinically significant [26,27]. Tissue components within the regions of interest were identified using color-based segmentation (positive pixel count algorithm v9.0 in ImageScope v11.2; Aperio, Vista, CA) similar to the methods of [15]. Initial image segmentation for each histopathologic slice was completed using a hue setting of 0.7 and a window setting of 0.35. These initial settings resulted in correct color segmentation for a majority of pixels into the required tissue components. Subsequently a test region for each slice was used to slightly modify the window and hue settings to minimize the number of pixels identified incorrectly (median absolute adjustment = 0.04; maximal adjustment = 0.08) due to variations in slice thickness and staining intensity. A single author determined the settings using visual assessment for each slice to allow for uniformity between slices regarding the correct identification of pixels as belonging to either the tissue cellularity or fibromuscular stromal matrix (FSM). These setting were verified for accuracy by the histopathologist who independently processed a subset (n = 10) of the cases for comparison. After segmentation the positive pixel count algorithm provided the total number of pixels within the region of interest, which were positive, or corresponding to tissue cellularity. This number was subtracted from the total number of pixels counted to acquire the total number of pixels within the region of interest, which were negative, or corresponding to FSM. By using pixel size these figures were converted into area. The combined area of cellularity and FSM was subtracted from the total region of interest area to calculate the luminal space area. The fractional area of cellularity and FSM was calculated as the ratio of the segmented area for each of the histological descriptors to the total region of interest (Fig 1). Luminal space corresponded to the difference between the total tissue area and the summed area of the pixels for cellularity and FSM.

### Statistical analysis

Medians and ranges were used to summarize continuous variables. Frequencies and percentages were used to summarize categorical variables. The Wilcoxon Rank Sum test was used to assess the relationships between DKI parameters and fractional tissue with disease aggressiveness (GS group) or tissue type (normal or PZ tumor). The correlations between fractional tissue components and median D_app_ or median K_app_ were evaluated using Spearman’s Rank correlation. Statistical analyzes were performed using SAS 9.2 software (SAS Institute, Cary, NC); p-values < 0.05 were considered statistically significant.

## Results

Overall, 20 subjects with significant PZ tumors were included (Table 1). The median time between biopsy and study MRI for the study subjects was 10 weeks (range 6–25 weeks). The median time between study MRI and prostatectomy was 10.5 days (range 0–34 days). The number of tumor ROIs (which was the same as matched normal ROIs) per patient depended on the size of the tumor and varied from 1–4 (mean 2). The tumor ROI size ranged from 3.1–365.4 mm^2^ (mean 92.0 mm^2^).

**Table 1:** Patient Characteristics.

Characteristic	
Patient age, years	64 (39–72) *
PSA level (ng/mL)	8.2 (2.0–14.6) *
Gleason score ^†^^ǂ^	
6 (3+3)	3 (15)
7 (3+4)	9 (45)
7 (4+3)	6 (30)
8 or higher	2 (10)
Pathologic tumor stage ^†^	
T2a	2 (10)
T2b	1 (5)
T2c	4 (20)
T3a	12 (60)
T3b	1 (5)

* Data are median and range (in parentheses)

^†^ Data are numbers of patients and percentage (in parentheses)

^ǂ^ Surgical Gleason score for largest peripheral zone tumor

PSA = prostate specific antigen

### Fractional tissue composition in PZ tumor and normal tissue

Table 2 displays the fractional tissue composition found in both PZ tumor and normal tissue. PZ tumors had a significantly greater fractional area of cellularity compared to normal PZ (p<0.0001). In addition, PZ tumors had a significantly lower fractional area of FSM compared to normal PZ tissue (p = 0.019) respectively, and a smaller luminal space (p<0.0001). Higher-grade PZ tumors had a significantly increased fractional area of cellularity compared to lower-grade tumors (p = 0.041). There was also a significant difference between higher-grade and lower-grade tumors in the fractional area of FSM (p = 0.011). There was no difference in the fractional area of luminal space for higher-grade compared to lower-grade tumors (p = 0.909).

**Table 2:** Fractional areas of tissue components for peripheral zone tumors and normal prostate.

Tissue Component	*Normal*	*Tumor*	* *
Cellularity	0.24 (0.10–0.40)	0.39 (0.32–0.61)	< 0.0001*
Fibromuscular stromal matrix	0.55 (0.45–0.65)	0.5 (0.32–0.55)	0.0192*
Luminal space	0.2 (0.11–0.33)	0.09 (0.06–0.21)	< 0.0001*
** **	***GS 3+4 or lower***	***GS 4+3 or higher***	* *
Cellularity	0.38 (0.35–0.47)	0.44 (0.32–0.61)	0.0279*
Fibromuscular stromal matrix	0.52 (0.45–0.55)	0.45 (0.32–0.51)	0.0049*
Luminal space	0.09 (0.06–0.15)	0.09 (0.06–0.21)	0.91

Data are median with range in parentheses

*Significant difference at p < 0.05

GS = Gleason score

### DKI parameters in PZ tumor and normal prostate

**Fig 2:**
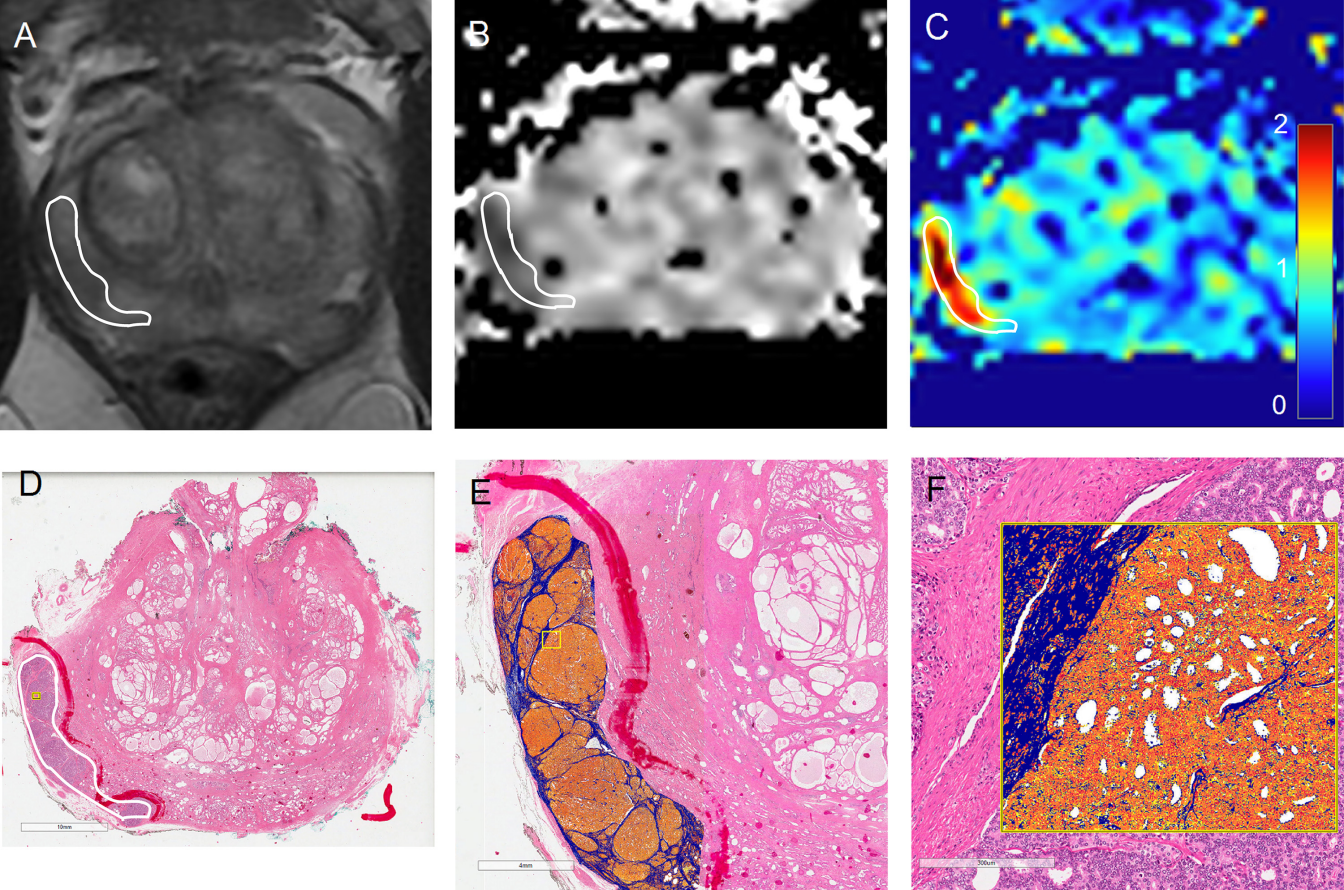
Image segmentation and DKI in a GS 4+5 right PZ tumor. MR imaging (A-C) and corresponding histopathological sections (D-F) from a 64 year-old man with Gleason score 4+5 prostate cancer (defined by white region of interest in A-D) and a pre-operative prostate-specific antigen (PSA) level of 6.7 ng/mL. **A:** Axial T_2_W-MRI shows a right peripheral zone tumor visible due to its low signal intensity compared to the surrounding peripheral zone. **B:** The axial D_app_ map (b-values of 150, 750, 1000, 1500 s/mm^2^) shows highly restricted diffusion within the tumor ROI with a median D_app_ of 1.08 x 10^−3^ mm^2^/s. **C:** Axial K_app_ map shows increased diffusional kurtosis in the tumor ROI with a median K_app_ of 1.28 (scale bar shown). **D:** H&E stained slide (x0.2 objective) shows a region of GS 4+5 tumor, which corresponds to the tumor ROI shown on MRI. **E, F:** Image segmentation (x0.5 and x10 objectives respectively) shows a highly aggressive tumor with the following fractional tissue composition: 0.61 cellularity; 0.32 FSM; 0.07 luminal space.

PZ tumors had a significantly lower D_app_ (median = 1.53 x 10^−3^ mm^2^/s, range 1.08–2.14 vs. 2.07, range 1.73–2.58; p<0.0001; Table 3) and a significantly higher K_app_ (median = 0.75, range 0.54–1.29 vs. 0.59, range 0.38–0.72; p<0.0001) when compared to normal PZ prostate tissue (Fig 2). Higher-grade PZ tumors (GS ≥ 4+3) had a significantly increased median K_app_ compared to lower-grade tumors (GS ≤ 3+4) (median = 0.87, range 0.66–1.29 vs. 0.71, range 0.53–0.88; p = 0.012). In contrast, higher-grade PZ tumors had a non-significant difference in median D_app_ compared to lower-grade tumors (median = 1.42 x 10^−3^ mm^2^/s, range 1.08–2.16 vs. 1.74, range 1.17–2.18; p = 0.200).

**Table 3:** DKI quantitative measures for peripheral zone tumors and normal prostate.

DKI measurement	*Normal*	*Tumor*	*p-value*
D_app_ (x 10^−3^ mm^2^/s)	2.07 (1.73–2.58)	1.53 (1.08–2.14)	< 0.0001*
K_app_ (unitless)	0.59 (0.38–0.72)	0.75 (0.53–1.29)	< 0.0001*
	***GS 3+4 or lower***	***GS 4+3 or higher***	* *
D_app_ (x 10^−3^ mm^2^/s)	1.74 (1.17–2.07)	1.41 (1.08–2.14)	0.20
K_app_ (unitless)	0.72 (0.53–0.88)	0.87 (0.66–1.29)	0.0122*

Data are median with range in parentheses

*Significant difference at p < 0.05

DKI = diffusional kurtosis imaging; GS = Gleason score

### Correlation between tissue composition and DKI

Comparison of the DKI parameters with tissue composition revealed a strong relationship with median D_app_ but not K_app_ in normal PZ tissue (Table 4). Median D_app_ showed a significant positive correlation with the fractional area of luminal space (ρ = 0.648; p = 0.002) as well as a significant inverse correlation with the fractional area of cellularity (ρ = -0.487; p = 0.029) for normal prostate tissue. There was no correlation with fractional area of FSM and median D_app_ (p = 0.840). In contrast, the median K_app_ of PZ tumors had a significant relationship with tissue composition, while the relationship with D_app_ was limited. Median K_app_ showed a significant positive correlation with fractional area of cellularity in PZ tumors (ρ = 0.50; p = 0.025) as well as a significant negative correlation with fractional area of FSM (ρ = -0.45; p = 0.049).

**Table 4:** Correlation between the fractional areas of each tissue component and the two quantitative DKI parameters.

	D_app_		K_app_	
	Rho	p-value	Rho	p-value
Normal prostate				
Cellularity	**-0.487**	**0.029**	-0.081	0.73
Fibromuscular stromal matrix	-0.049	0.84	0.336	0.15
Luminal space	**0.648**	**0.002**	-0.233	0.32
Peripheral zone tumor				
Cellularity	-0.415	0.07	**0.499**	**0.025**
Fibromuscular stromal matrix	0.199	0.40	**-0.445**	**0.049**
Luminal space	0.228	0.33	-0.128	0.59

## Discussion

This prospective study assessed the fractional tissue composition of normal tissue and peripheral zone tumor within the prostate by using digitalized histopathology acquired following prostatectomy. Tumors had significantly increased cellularity, as well as decreased fibromuscular stromal matrix (FSM) and a decreased luminal space compared to normal tissue. Furthermore, in more aggressive tumors, the cellularity was further increased and the FSM decreased without a significant change in the luminal space. These measurements of tissue composition were subsequently correlated with DKI showing a positive correlation between the median K_app_ and cellularity and a negative correlation with FSM in tumours. Median D_app_ had a positive correlation with luminal space and a negative correlation with cellularity in normal tissue.

The differences between the correlation of K_app_ with tumor tissue and the correlation of D_app_ with normal tissue could be explained by the biophysical nature of water diffusion. It is proposed that DWI largely measures water diffusion in the extracellular compartment [28]. A significant portion of this is present within the luminal space in normal prostate tissue, and studies that have investigated D_app_ and percentage luminal area have found a significant positive correlation between these two parameters [15,29]. In contrast to luminal space, which largely represents an area with free water diffusion, the FSM and cellular regions of tissue have increased tissue complexity creating barriers to the free diffusion of water [28]. The results presented here support the hypothesis that DKI is probing the heterogeneity of tumor tissue, which contrasts with the more homogeneous normal tissue. This is further supported by a previous study that found a restrictive effect of cellularity and FSM on the diffusion of water in prostate tissue using high field microimaging of *ex vivo* prostate samples [30].

In this study, the positive correlation between median K_app_ and tumor cellularity, in association with the negative correlation with FSM, provides a possible biological basis for the significant difference in median K_app_ detected between lower-grade and higher-grade PZ tumors. A retrospective study by Rosenkrantz *et al*. found that K_app_ was significantly increased in the biopsied prostate regions with GS 7 or 8 tumor, compared to those with GS 6 [17]. A significant difference for DKI parameters obtained from tumors with GS 6 compared to GS 7 or higher has been shown by other retrospective studies [18–20, 31]. This ability to accurately differentiate GS 6 disease could be useful in the setting of active surveillance. The prospective study presented here expands upon this work correlating DKI with aggressiveness and, importantly, compares the results with the tissue composition derived following prostatectomy. Instead of using a cut-off of GS 7 or higher, as has been undertaken in other studies, this study subdivided GS 7 disease to further investigate the underlying biological basis for the change in diffusion parameters. The increase in tissue heterogeneity that occurs as the tumor shifts from Gleason grade 3 to 4 could explain why a significant difference in K_app_ was detected between lower-grade and higher-grade disease in this study. Studies have previously shown the clinical significance of GS 3+4 versus GS 4+3 [26,27] and the International Society of Urological Pathology recently proposed a new grading system, which more clearly makes this distinction [32]. This study provides evidence that K_app_ offers a potential measure of tissue heterogeneity and cellularity, which could be used to discriminate higher-grade from lower-grade tumors, and further research investigating its possible role is warranted.

This study has also demonstrated that both the apparent diffusion (D_app_) and apparent kurtosis (K_app_) showed a statistically significant difference between PZ prostate cancer and matched normal prostate. Previous studies that have investigated the ability of DKI to detect PCa lesions have found mixed results. In a retrospective study of 47 patients using biopsy results as a reference standard, Rosenkrantz *et al*. [17], found that K_app_ showed a greater sensitivity (93.3%) for differentiating cancerous sextants from benign PZ compared to either ADC (78.5%) or D_app_ (83.5%) however this was associated with a decreased specificity for K_app_ (70.0%) compared to ADC or D_app_ (81.4% and 82.9% respectively). In addition two other smaller studies did not demonstrate a clear improvement in tumor detection using DKI [33, 34]. The results of this study suggest that the principal benefit for DKI, K_app_ in particular, is regarding its association with tumor aggressiveness and highlighting regions or tumors with more aggressive tissue characteristics.

Assessing tumor aggressiveness by combining measurements from both histopathology and non-invasive imaging is important for accurate disease characterization and treatment [8,9]. Furthermore, non-invasive imaging can be used to more accurately guide tissue sampling of the prostate, and thus further improve the characterization of the tumor [35]. The results from this preliminary study show that DKI could aid in the direct sampling of regions with increased aggressiveness or tissue heterogeneity, and therefore could assist in the histopathological assessment of the disease in the future.

Our study has some limitations. First, this was a preliminary study with a small sample size and we were limited to univariate analysis; despite this, clear statistically significant results were demonstrated and future evaluation in a larger patient group is required. Second, our study was limited to PZ tumors because there were few TZ tumors in this cohort. Third, accurate correlation between imaging and histopathology is methodologically challenging: while every effort was made to correlate histopathology and imaging, some mis-registration may have occurred. Fourth, this study did not specifically evaluate the presence of neovascularization either through the DKI parameters or the tissue composition work. Such evaluation could be useful in further studies. Finally, the Gleason score was used as a surrogate of clinical outcome as a well established predictor of clinical outcome after radical prostatectomy and radiation therapy; longitudinal studies are required to directly correlate these imaging findings with clinical outcome over many years.

## Conclusions

In summary, this study builds on previous work in the field of diffusion-weighted imaging of the prostate and, in the setting of a prospective study, relates DKI to tissue structure using histopathology acquired from prostatectomy. By comparing DWI parameter maps with histological sections, this work has demonstrated the structural changes in tissue composition that occur in normal prostate tissue and in tumor, and between tumors of different grade. Using this methodology, this study has provided an explanation of the biological basis for the metrics derived from DKI. The two parameters obtained—D_app_ and K_app_—provide complementary information: D_app_ probes the relatively homogeneous luminal space and cellularity present in normal prostate and was significantly lower in PZ tumor compared to normal prostate; K_app_ reflects the increase in cellular heterogeneity present in PZ tumors and was significantly higher in more aggressive tumors compared to less aggressive ones. This distinction in GS is important clinically: accurate non-invasive evaluation of the tissue structure and tumor grade within a suspected prostate cancer could help to determine which men may require a biopsy, and could help to stratify patients into those suitable for active surveillance and those who should receive focal therapy or surgery.

## Supporting Information

S1 Table Study Data Set. (XLS)
